# Assessing the impact of a 6-year health sciences enrichment program for underrepresented minority youth on healthcare workforce diversity, career path, and public health

**DOI:** 10.3389/fpubh.2023.1244593

**Published:** 2023-10-12

**Authors:** Oscar B. Kohut, Zhiru Wang, Ronald R. Sanchez, John C. Rausch, Andy Nieto, Mara M. Minguez

**Affiliations:** ^1^Division of Community and Population Health, New York-Presbyterian Hospital, New York, NY, United States; ^2^Department of Pediatrics, Columbia University Irving Medical Center, New York, NY, United States

**Keywords:** underrepresented minority youth, health sciences education, youth development, pipeline program, qualitative program evaluation, constant comparative analysis, grounded theory

## Abstract

**Background:**

Improving the quality of care for a diverse population requires a diverse healthcare workforce which necessitates high educational attainment among underrepresented communities. Programs aimed to address healthcare workforce diversity gaps also serve as a public health intervention by offering avenues to improve the health of local communities by providing students with the knowledge and skills to promote healthy behaviors, foster scientific literacy, and inspire future public health professionals - who in turn serve their local communities to advance health outcomes. We interviewed alumni of the New York Presbyterian Hospital Lang Youth Medical Program (LYMP), a high school health sciences mentoring and enrichment program for underrepresented minority youth in Upper Manhattan, from graduating classes between 2012 and 2021 to explore their perspectives on what aspects of the program had the most impact on their academic and career paths.

**Method:**

This is a qualitative study using in-depth, semi-structured individual interviews. All interviews were analyzed using the constant comparative method for developing grounded theory, following a convenience sampling method.

**Results:**

106 codes were organized into 24 themes, which were further arranged into 4 topic areas: demonstrated program success, intangible program drivers, improvement opportunities, and barriers to program participation. Topic areas captured participants’ perspectives on how the program is designed to foster an environment of personal, academic, and professional development; ways aspects of the program organically worked together to provide unanticipated positive facilitators; opportunities for program improvements, and external factors that influenced decision-making.

**Conclusion:**

Through this study, we found that the LYMP had a positive influence in helping participants set and achieve personal, academic, and professional goals. Alumni reported activities and experiences offered by the program that foster key youth development constructs linked to healthier and more resilient communities. Importantly, the vast majority of participants described how the synergism between program features, staff support, family involvement, and professional development and networking created an environment of achievement that went beyond the scope of the program design. Findings from this study offer a blueprint for other organizations to craft a similarly successful enrichment program that improves health outcomes, reduces health disparities, and promotes overall population health.

## Background

### Problem statement

There is a need to increase the proportion of underrepresented minority groups in the healthcare workforce in order to provide high quality and accessible care for a diverse patient population (1, 2). Differences in access and quality of care are connected to the lack of diversity in the healthcare workforce, with African-American doctors and Hispanic doctors each constituting less than 6% of physicians in the workforce (3). Trends in workforce capacity among Underrepresented Minorities (URMs) reflect this need for greater diversity. For instance, national data show that while the numbers and proportions of URM nurses significantly increased to approximately 19% in 2018 and 2019, the URM workforce continues to be underrepresented compared to the 31.6% in the U.S. population due to unequal educational and professional opportunities (4). Greater diversity in the healthcare fields has helped increase access to care for underserved populations, improve access to healthcare, improve the cultural competence of the workforce, and enhance the educational experience of students in the healthcare workforce (5).

In addition to increasing workforce diversity, healthcare pipeline programs have shown numerous positive effects for students, like increased standardized test scores (6), increased social capital (7), and meaningful long-term professional impact (8, 9). Black and Latinx students are at an educational disadvantage compared to public schools in more affluent areas with access to more resources (10). Studies show that elementary and secondary schools with large numbers of Black and Latinx students are less likely to have experienced teachers, advanced courses, high-quality instruction materials, and adequate facilities (10). New York-Presbyterian Hospital/Columbia University Irving Medical Campus (NYPH-CUIMC) is located in New York City Department of Education District 6: Washington Heights and Inwood, where 92% of students identify as Black or Latinx (11). The most current publicly available state exam data provides results from 2015 to 2017, showing that 56% of Black and Latinx high school students in this district passed the living environment Regents exam, compared to 74% for all other students in New York City (13).

### The Lang youth medical program: a response to diversifying the healthcare workforce

In response to the rising need to improve diversity in the healthcare workforce and academic achievement in the local school district, NYPH-CUIMC founded the Lang Youth Medical Program (LYMP) in 2003 to provide students from those communities with the opportunity to nurture and explore their interest in the health sciences. Based under the umbrella of NYP’s Division of Community and Population Health, and led by Columbia faculty and NYP staff, the program provides high-quality health science, public health, and professional development programing to students in the Washington Heights and Inwood area through hospital-based mentorship and internship opportunities (12).

With NYPH-CUIMC as a resource for mentorship and hands-on medical experience opportunities, students at the LYMP are provided with quality professional and personal development to help them achieve their goals and bridge the educational gap that students face. LYMP provides students who aspire to attend a four-year college the opportunity to explore careers in healthcare and become leaders in their community. While the LYMP aims to inspire students to achieve their academic and professional goals, the program also focuses on providing students with a rich and vibrant sense of community that creates life-long lasting relationships amongst peers, and between scholars and mentors.

### Program structure

The Lang Program aims to educate young scholars by providing them with a tailored academic curriculum (see Appendix 1), goal development, and support services. The program has several long-term goals: first, to implement a program following a logic model (see Appendix 2) that is purposefully designed to provide students with science education, health career exploration and counseling, college preparation, personal and professional development, staff development, financial support, networking, family support, and alumni engagement; secondly, to develop a program that can be replicated by other medical centers to increase the number of minority scholars who graduate with postsecondary and professional degrees, and are committed to pursue a career in the health sciences; lastly, to increase the number of minority students who return to their communities to work as health care professionals.

Each LYMP cohort consists of 12 to 15 high-achieving seventh graders attending a middle school in the Washington Heights-Inwood school district. Students in sixth grade applying for the program must have a grade point average of 80% or greater in all subjects, an interest in exploring careers in healthcare, and the ability to make a six-year commitment to the program. Scholars attend program classes on Saturdays during the academic year and weekdays during the month of July. LYMP’s six-year curriculum is composed of five focus areas: health careers exploration, science enrichment, high school and college application, mentorship opportunities, and psychosocial support (Table 1).

**Table 1:** Lang youth medical program curriculum.

Program focus areas	Program activities and learning objectives
Health careers exploration	Program activities Hospital tours Career panels Clinical rotations Paid internships in clinical and non-clinical health fields Personal and professional skills development workshops Learning Objectives Comprehend the intricacies of various hospital departments, functions, and workflows, gaining insights into healthcare professionals’ collaborative role in patient care. Explore diverse healthcare pathways, interact with seasoned practitioners, and identify essential skills and qualifications for different roles. Understand the daily responsibilities of clinical and non-clinical positions. Cultivate leadership attributes, teamwork capabilities, and ethical decision-making skills, all within the healthcare context.
Science enrichment	Program activities Classes on the structure and function of the human body systems Lectures on disease pathology and prevention Case study round tables Global and public health curriculums Learning Objectives Gain proficiency in understanding human anatomy, functions, interdependencies of body systems, and identify disease implications Study disease causes, progression, and preventive strategies Enhance critical thinking by participating in patient case analysis discussions Understand health disparities, global challenges, and community involvement
High school and college application	Program activities College application assistance SAT tutoring College campus tours Alumni career development Learning Objectives Develop skills to create impactful college applications that showcase their achievements, aspirations, and unique qualities Provide tailored instruction and strategies aimed at elevating students’ SAT scores. Exposure to diverse college environments and facilities, fostering informed decision-making to make well-matched choices regarding their higher education paths. Develop skills aimed at advancing long-term professional objectives
Mentorship opportunities	Program activities Exposure to and guidance from healthcare professionals, and students from undergraduate, graduate, and professional schools Learning Objectives Foster healthcare profession understanding via direct interactions and mentorship with diverse professionals and students across educational levels.
Psychosocial support	Program activities Connecting students and families to community health care and wellness services Learning Objectives Connect students and families to vital community health services, enhancing resource awareness, utilization, and comprehension. Promote community well-being by facilitating access to essential healthcare and wellness resources.

As of 2021, 85 students are enrolled in the program. The male-to-female ratio is approximately 1:1, with 70% of students identifying as Latinx.

The program’s first cohort of LYMP alumni graduated in 2009. Since that time, the alumni cohort has grown to 140 alumni. The LYMP alumni profile currently consists of 52 undergraduate students, 13 graduate students, and 40 professionals that work in a diverse range of fields including business, education, and healthcare. Eligible alumni may receive up to $6,000 in scholarships throughout their undergraduate careers for academic-related expenses, which is fully funded by LYMP.

Scholars who graduate from the program are invited to attend two annual LYMP alumni reunions where they are able to connect with old and new peers, network with other alums and professionals, as well as become mentors to the newest class of high school program graduates. A newsletter is disseminated quarterly with details on employment, internship, and other resources for alumni to further advance their professional and career interests.

Alumni have also returned as hospital volunteers, summer and academic per-diem LYMP advisors, and full-time employees of NYPH-CUIMC. Currently, there are five alumni working at NYPH-CUIMC within the Ambulatory Care Network, two of whom are registered nurses and one of whom was hired full-time as a coordinator for Lang.

### Health profession pipeline programs and research models

The importance of health profession pipeline programs for underrepresented students at the middle and high school level plays an important role in determining the future healthcare workforce. Research has shown that minority healthcare workers are more likely to practice in underserved communities (14). Additionally, a growing body of evidence indicates that diversity in professional health education can improve cultural competence and reduce implicit bias, which ultimately improves health outcomes and can be a driving force to reduce healthcare disparities (15, 16). Although diversity in the health care professions is increasing, minority representation in health care occupations remains substantially below the diversity of the general population (16, 17). Factors contributing to a lack of diversity may include inadequate secondary education, limited financial support, increased educational levels required for entry into some professions, and lack of mentorship (16).

Evidence on career education and counseling, professional and research experiences, and meta-analysis on other educational strategies to improve academic outcomes among young students is generally positive (18–20). Services provided in these pipeline programs include rigorous courses, career counseling, financial support, college field trips, internships and shadowing experiences in professional settings, psychosocial support, and job placement (20, 21).

Among minority students, health professions pipeline programs have demonstrated to increase the number of minority students who go on to pursue higher education in the sciences (21). Research shows that health professions pipeline programs are key drivers to whether students attain the degrees and skills to grow the diversity of the health professions, whether minority health care workers pursue high-need specialties or work in underserved populations, and whether these students have been empowered to develop the skills to advance health equity efforts (19, 22–25).

Programs that develop capacity building and empowerment have demonstrated an effect to increase the knowledge and skills youth need to succeed (26). The design of the Lang Program to develop specific skills and competencies, build character, and provide supports tailored to student needs is aligned with other prevailing youth developmental frameworks: The Positive Youth Development, and the Development Assets frameworks (27–29).

Youth developmental models focus on developing the psychological, behavioral, and social characteristics needed for success, while also considering the structural supports needed to enhance the impact of external facilitators and reduce those of external barriers of achievement. Programs that integrate this model aim to provide opportunities to participate in meaningful activities with real-life applications, have high expectations, encourage deep positive relationships and connections with adults, peers, and the larger community, and have an environment of care and support (28, 29).

The Development Assets framework provides further guidance on youth development by focusing on young people’s successes rather than failures, and viewing them as resources (27). The constructs within this framework directs attention onto strengths young people have with a belief that programs providing social support and empowerment, boundary and expectation setting, constructive use of time, encouraging a commitment to learning, and developing social competencies, positive values and identity, results in positive outcomes (27).

The constructs within these frameworks are cultivated throughout the program by design, such as courses in STEM, activities centered around interpersonal and professional skills, financial and family support, and providing an opportunity for scholars to develop deep, long-term, positive relationships with peers and professionals.

### Research aims

This evaluation is an opportunity to further understand how a hospital-based youth mentoring and education program can influence the life trajectory of underrepresented minority students in pursuing academic and career paths in the health sciences. As a qualitative evaluation, we interviewed alumni to explore their perspectives on what aspects of the program had the most impact on their personal and professional choices and to offer areas of improvements.

This study aims to answer the following research question: How has the Lang Youth Medical Program impacted the academic and career paths of its alumni?

## Methods

The Columbia University Irving Medical Center IRB approved this study’s procedures.

### Participants and enrollment

The setting for this research was NYPH-CUIMC, which provides funding for the Lang Youth Medical Program. Participants were determined to be eligible for recruitment if they graduated between 2012 and 2021 (*N* = 152). The LYMP administrator sent eligible alumni emails to participate in the study and register for an interview. Alumni from graduating classes in 2014, 2016, and 2019 had a slower rate of participation, thus, text messages from the LYMP administrator were also sent to these alumni to ensure participation from all graduating years. As eligible alumni volunteered to be interviewed on a rolling basis during the 5-month study period, this study followed a convenience sampling method.

During the online registration process, the alumni’s name, email, graduating year, and phone number were collected. Upon registration, alumni received information via email by two study team members, OK or ZW, regarding their selected interview time and date, the purpose, and design of the study. Twenty-four hours prior to the interview, study participants received an email reminder of their appointment by OK and ZW.

Participants were considered enrolled into the study upon providing verbal consent to the interviewers prior to beginning the interview. Participants consented to be audio-recorded only and were able to skip any question they did not want to answer or end the interview at any time. Enrolled participants were compensated $50 for their participation in answering any or all questions.

### Interviews

The interview guide was developed through an iterative and collaborative process by the research team. *A priori* themes were generated by taking into account the design of the program (e.g., inputs, program activities, and desired outputs) would influence alumni perspectives (Appendix 2). To a lesser extent, *a priori* themes were also informed relevant developmental frameworks, such as the UNICEF Conceptual Framework for Measuring Outcomes of Adolescent Participation (26), and The Positive Youth Development, and the Development Assets frameworks (27, 28), and semi-structured, open-ended questions were constructed that would allow participants to describe their perspective in detail, and enabled the interviewers to probe responses for more information.

In depth, semi-structured individual interviews, lasting approximately 1-h each, were conducted by OK and ZW using Zoom® (v.5.10.6) for audio-recording and closed captioning. All participants were audio-recorded only. Transcripts were de-identified and loaded into Dedoose® (v.9.0.46) for coding and data analysis. Data files were stored in secure locations, accessible only to the research team per IRB protocol.

Interviews began with questions about participants’ age, gender, and racial/ethnic identity. Next, interviewees were asked about their current academic and professional endeavors. Questions then focused on the alumni’s time in the program, with particular interest on the elements of the program that they found to be most impactful to their personal, professional, and academic growth. Additionally, part of the interview was dedicated to understanding the program’s impact on how alumni have been able to cope with the changes that had occurred in their life as a result of the COVID19 pandemic. Interview questions concluded with an opportunity for alumni to provide input on which parts of the program were the most useful and which could be improved.

### Analysis plan

Throughout the data preparation and analysis process, principles from the constant comparative method of qualitative data analysis were applied to develop a grounded theory, whereby the uniqueness of each participant’s experience was carefully considered while exploring commonalities of experiences across all participants to craft codes, themes, and topic areas (30). To reach saturation, a minimum goal of 30 interviews was determined by the research team to be the most feasible and reasonable, considering constraints on time and resources, as well as the study being a first-of-its-kind exploratory analysis. Guidelines for similar qualitative studies suggest a sample size between 20 and 30 interviews (31, 32).

Interviews and data collection occurred over a 5-month period and were conducted by OK and ZW. RS supervised OK and ZW during the data collection and coding process. Transcripts were coded in small batches and OK, ZW, and RS met regularly to revise, add, and delete codes, and ensure the final codebook contained codes that were applied uniformly. Once the codebook was finalized, codes were reviewed for their thematic application by all members of the team. JR and MMs led the organization of themes into the final topic areas. Development of the topic areas followed the same approach as the code and thematic process: use of multiple thematic perspectives to understand the data and reach a consensus on the perspective of each topic area. All members of the research team hold graduate or professional degrees, including training in qualitative research methods.

## Results

Table 2 shows participant characteristics. Most participants were ages 18 to 24 (*N* = 21 [78%]). Lang Program scholars graduating in 2018, 2019, and 2021 represented 52% (*N* = 14) of those interviewed. Of the 27 study participants, 67% (*N* = 18) were female, and 52% (*N* = 14) identified as Latinx.

**Table 2:** Participant’s characteristics (*N* = 27).^*^

Graduating year *N* (% Total)		Age group *N* (% Total)		Gender *N* (% Total)		Race/ethnicity *N* (% Total)	
2012–2014	4 (15%)	18–24	21 (78%)	Male	8 (30%)	White	2 (7%)
2015–2017	9 (33%)	25–26	6 (22%)	Female	18 (67%)	Black	5 (19%)
2018–2021	14 (52%)			Non-Binary	1 (4%)	Latinx	14 (52%)
						Asian Pacific American Indian	3 (11%)
						Multiple	3 (11%)

* As of time of the interview.

Twenty-seven alumni were interviewed, generating 106 codes. Following the constant comparative method, codes were organized into 25 themes, which were further arranged into four topic areas: demonstrated program success, intangible program drivers, improvement opportunities, and external facilitators and barriers to program participation (Table 3). Although several prior themes were hypothesized, the research team found many more new codes, themes, and topic areas emerged from analysis.

**Table 3:** Topic areas and themes.^*^

Topic area	Themes	Topic area description
Demonstrated program success	Academic skills Coping skills Interpersonal skills Professional skills Program academic content Program activities Developing goals Intended academic choice Pursued a health career Research experiences post-LANG Perception change Program support	Participants described the ways by which the program fostered an environment of knowledge and skill building, including how the program helped them think and plan their academic, personal, and professional goals; how the program provided supports that reinforced their pursuit of a health career, or positively changed their perception on a career in the health-sciences. Themes reflect program design features that have been proven in the literature to help young people achieve their goals.
Intangible program drivers	Attitude changes Developing a sense of self Did not pursue a health career Lang values Memorable/impactful activities Program satisfaction Relationships Trade-offs Transferable skills	Participants described the way by which the program developed their intrinsic motivation for change and growth, exemplified program values, developed life-long skills, had a long-lasting impression, and may have changed their attitudes toward a specific career or health profession. Themes reflect participant’s perspectives on how the synergism between program features, staff support, family involvement, personal and professional networking, and post-graduation activities have impacted their current lives.
Improvement opportunities	Program Limitations Recommendation	Participants gave specific critiques regarding program commitments, activities, and recommendations to improve programming around career choices, program activities, and outreach efforts.
External facilitators and barriers to program participation	External drivers Financial hardship	Participants describe ways that external factors influenced their decision-making process around academic, personal and professional choices.

* Quotes presented herein were pruned for conciseness and readability, while preserving their meaning.

### Demonstrated program success

Themes within this topic area reflect program design features that reinforce the development of important constructs proven in the literature to have demonstrated success among other programs seeking to help young people achieve their academic and personal goals (26–28). Participants described the ways by which the program fostered an environment of learning and skill building, including how the program helped them think and plan their academic, personal, and professional goals. The impact the program has had on study participants’ academic and career paths is made evident in their description of how the program provided support that strengthened their pursuit of a health career, or positively changed their perception on their ability to pursue a career in the health-sciences.

Aligned with the design of the program, participants provided specific examples of the types of training they received during their time in the program, which included academic, coping, interpersonal and professional skills. Participants also described receiving rigorous academic content and coursework, participating in extracurricular program activities, and developing short- and long-term goals.

#### Academic skills

Participants recalled exercises in essay writing, health literacy, lab, scientific method, and test prep as important academic skills. Of the academic skills learned, health literacy skills had the most profound and long-lasting impact on participants. Health literacy skills are cultivated during the Lang Program by teaching health information and how that information can be used to make good health-related choices. Participants said that this was an important and useful experience:

…for example…one of my friends in high school had…a pregnancy scare, she was super freaked out… [I said] …, “Well, you can get tested first before we freak out.” I knew this from Lang [sexual education class], so I was giving her counseling, and I told her not to freak out…I think having that knowledge was helpful for me to not only make good decisions myself, but also help other people make decisions that were right for them. …I think they prepared us very well for life. (P5)

#### Coping skills

Scholar’s transition from childhood to adolescence to early adulthood during their time in the Lang Program. One participant recall discussing “life lessons” (P5), covering topics in “reproductive health, substance use, and other factors that affect adolescent life…,” which “helped [them] maintain [their] composure throughout adolescence.” (P5)

Building resiliency and stress management skills are other coping mechanisms participants mentioned learning during their time in the program:

We would meet up with our mentors and talk about any stresses that we had, any struggles we saw the [medical staff at their internship] had…and they would give us advice and we would have classes on stress relief. (P19)

Lang really taught me…the best skills to use when feeling overwhelmed. They've really helped me figure out what works best for me and how to do things that work for me, but they also allowed me to understand that things are trial and error, and not everything is going to work out in the first shot. So, there's always different things to try out to see how they work. (P1)

#### Interpersonal skills

Practicing communication and empathy were important interpersonal skills training experienced by alumni:

The program definitely helped [me] with communicating with…different types of people, since we were interacting not only with doctors and those within the medical field, we were also interacting with our peers daily, we were interacting with those in grades above us and below us, and we were speaking with our advisors. (P26)

One thing I believe Lang really emphasized…and it was…key to the program was empathy. That's something that I never really thought about, prior to going into the program, but Lang made that really important and really emphasized it. So now, every time [I am] interacting with someone, I always try to look at things from their point of view. And that allows me to get a deeper understanding of their situation…and really understand that I am able to help the individual. So, I think Lang has really influenced how I interact with people in the sense that I have tried to look at their perspective first before I try to respond. (P24)

#### Professional skills

Participants described professional skills, which are designed to meet the demands of professional and work settings, such as essay writing, interviewing, leadership, problem solving and critical thinking, setting boundaries, and other practical skills (e.g., presence and appearance, note-taking, and providing productive contributions to a discussion). One participant describes the professional expectations of the program:

So, I remember every Saturday, we would have to go dressed up to program with slacks and a nice shirt. That was one way they helped us in our professional life because you know since we were young, they expected us to act a certain way at such a young age, to prepare us for the future. So, they helped us build character, act professionally… work, and organize our time. I remember having to do those classes [where] they would build a schedule for us. They would give us time to organize our days and that helped us grow into the professionals that we are today. (P19)

Another participant described how the program went beyond academic training to cultivate professionalism:

It’s an academic program where you’re learning about medicine… But it’s so much more than that…. You’re not just learning about medicine, you’re learning how to…talk to each other, you’re learning about how to show yourself off in a professional setting, you’re learning responsibility…You know, small little things like that, they help reinforce what you’re going to need in the future. And they don’t seem like they will, but they definitely will. (P18)

Exercises in leadership skills were paramount in developing one participant’s professional abilities:

…leadership skills that were very helpful having learned in Lang… for instance…not always being on top of the situation but rather more often listening and letting other people talk, instead of kind of me [being] a narcissistic leader… (P2)

This alumni describes how the Lang Program motivated them to take initiative in their ability to communicate their thoughts as a leader:

I think taking initiative, leadership, and…I would say…speaking your mind. I [now] feel like I’m never afraid to say what I need to say, or just say my opinion, and I think that’s one thing I feel like Lang has definitely prepared me for… I think speaking my mind. I’m never afraid to do that, and I think throughout college that has helped me, Lang has helped me develop that strength. (P26)

#### Program activities and academic content

Study participants listed case studies, exposure to health sciences courses, and dissections as impactful academic training. Program activities mentioned by alumni included class trips to colleges and healthcare expositions, panelist presentations, hands-on experience and internships in a hospital setting, and courses dedicated to college applications, SAT prep, and college readiness.

#### Developing goals, intended academic choice, pursued a health career, and research experiences post-Lang

During the interviews, participants described how the Lang Program provided structure and guidance to help them develop goals related to their academic and professional aspirations. One participant stated that being exposed to healthcare careers allowed them to “[understand] that the medical industry and the health field is not just having to pursue medicine. It can be things like public health, which we learned in the program” (Participant 25). Another participant shared that they “started to realize different things about the medical field and that’s what Lang was for, you know, to really open up that pathway for us to see the real medical field and for us to know what it’s like. So, it really helped me find what I wanted to do, which is psychology.” (P1).

Participants also described how the program activities reinforced what major they would pursue in college or informed that decision. Eleven alumni pursued a STEM major at the undergraduate level. Another four alumni continued to pursue a graduate education across various academic fields. Additionally, four participants engaged in scientific research at the postsecondary or professional level after graduating from the program.

Alumni that chose to pursue a career in healthcare found that their time during the program helped them transition directly into a profession that was consistent with career goals they established during the program (*N* = 13), provided the foundation to pivot into an adjacent health career (*N* = 9), or gave them guidance on the best intermediate step(s) to achieve their LYMP career goals (*N* = 9).

#### Program support

A number of participants described how the program is designed to provide tailored support to meet their mental, well-being, and financial health needs. Additionally, respondents commented on receiving mentoring and guidance from other alumni, peers, program staff, or professionals engaged with the program outside of the programmed services. Alumni also reflected on the program’s flexibility to accommodate their personal situations. Particularly, five (5) alumni recalled receiving support from program staff or individuals associated with the program to help them through COVID19 related issues.

#### Perception change

A majority of study participants provided specific examples on how their perception of the health professions changed as a result of the various program activities. One respondent describes her time shadowing a nurse in a family health center as “getting a chance to see what those [nurses and doctors] actually do. I would see how they assessed the patients, what medications they give, [and] how they interact with patients. The program opened my eyes even more to what a day in their shoes … and … their profession is really like.” (P19) Another student spoke on how the program showcased “different areas of healthcare, and how they intersect with one another” (P20), and that “the program was really set on making sure to include the …business…and…research aspects of [healthcare]. That’s something I would not have realized if it wasn’t for the program. I did not even think of it as part of the healthcare [field] until I entered Lang.” (P20)

### Intangible program drivers

#### Program satisfaction

23 alumni expressed their gratitude and satisfaction toward the Lang program as it gave them meaningful experiences and empowered them to pursue healthcare professions. Several participants believed the Lang program was an “eye-opening experience” (P3) and they “would definitely do it again.” (P7) One alumni found the Lang program to be a life-changing experience:

It changed my life. Even though I don’t think that I’m going to work in a hospital, it definitely impacted my life for the better… (P12)

Some participants made specific comments regarding the program’s academic and professional resources, such as resume building, essay writing, SAT prep, networking, and shadowing opportunities:

I would not have known how to write a resume, I wouldn’t have been able to write my…college essay [without the Lang program] …they helped me so much…I definitely wouldn’t have been able to get into Hunter [College] without them. (P18)

[Lang was] always introducing us to different doctors, to different health care professionals. And I think just because most of my childhood was in the hospital, that made the transition [to the clinical setting] a lot easier.” (P17)

One alumni suggested that “the program made [her] think that it was a lot more attainable or just easier to break into or to dive into [the medical workforce]. That essentially [means] that if you wanted it, you could just do it.” (P10) The same idea was supported by another alumni by saying: “the program … shows you those opportunities at a young age and so you can visualize it and see [it].” (P12)

#### Relationships

The Lang program fostered strong relationships through its engaging program activities, including internships, curriculum, and workshops. 20 participants talked about their peer, staff, and professional connections and their essential roles in “strengthening how youth contribute to the communities they live in” (26). One alumni said that “you are not just learning about medicine, you are learning how to sit and talk to each other” (P18). The strong connections these alumni developed throughout the program have lasted beyond the program:

We are also super close. One of our classmates had a baby a couple months ago and we all went to her baby shower…I texted [the Lang Medical Director] a month ago to let her know I have officially declared general surgery [as my specialty of interest]. Joey was the former alumni relations and I still chat with him.” (P17)

I am still in touch with the dental internship [connection]…And I'm also in touch with the Lang coordinators. (P15)

#### Trade-offs

To better understand the program barriers, our research team specifically asked for trade-offs alumni made to participate in the Lang program. Many participants described time commitment as a major barrier: “It was a very long program; it was 6 years… a lot of our time was taken up” (P26). Time conflict is another common obstacle as students always have multiple commitments: “I was in middle school, and I was on the soccer team. I remember and there were some games that I wasn’t able to go to because of the commitment that I had with Lang” (Participant 24). Another alumni spoke about how they had less time to hang out with friends because “[on] Saturdays, we have to go to Lang and in the summer, it’s kind of intensive and it’s almost like school where you are going almost every day” (P27).

While 21 out of 25 participants talked about the trade-offs they had to make, 12 of them also found the “sacrifice” to be worthy and “never thought it was too much of a tradeoff” (P6).

#### Transferable skills

The Lang program has focused on soft skill development by engaging students in a variety of social, academic, and professional activities. Some skills brought up by participants include networking skill, critical thinking skill, cultural competency, health communication skills, learning/cognitive skills, adaptability, organizational skills, presentation skills, public speaking skills, and time management skills.

19 participants believe Lang taught them the essence of networking and encouraged them to never “be afraid to reach out” (P22):

They pushed me to interact more with professors and teachers to have a better relationship with them because not only is it useful to you, but in the future, they could be the ones writing you a recommendation or telling you of certain opportunities that are there because you built a good relationship with them. (P13)

Many participants believe they develop strong communication, public speaking, and presentation skills through Lang-related events:

I have to mention the yearly expos… I think that definitely helped with my public speaking skills, and just speaking with different people. (P10)

Those presentations [at the Expo] were the reasons why I became more confident in public speaking or even classroom presentations. (P20)

12 participants mentioned becoming better at time management due to Lang projects, events, and courses, and they learned to “organize things properly [to] get everything done” (P23). Additionally, some alumni believe they developed critical thinking and learning/cognitive skills through the Lang curriculum and case studies:

I think [Lang] really helped me to grow in my critical thinking [ability] and learning to connect information even if it didn't seem related, you know, pulling out meaningful information from experiences and other things that I know and relating it to what I'm learning. (P27)

I guess other skills that I [learned was] cognitive thinking applications or being able to see parallels. And what does it mean to me or seeing my parallels and memorization. (P8)

One participant believed the program helped them develop cultural competency: “if I wasn’t for Lang, it would be a little bit more difficult for me to give my patients culturally competent care” (P4).

Adaptability was another important skill mentioned by participants where they became better at adapting to unexpected situations, such as the COVID pandemic:

So, I'm very used to Lang, used to adapt in and make the best of the situation…I'm sure that definitely had an impact in how I approach the changes that were occurring during the pandemic. (P24)

#### Attitude changes

Attitude changes were defined as changes in attitude toward a specific career or health profession that may have led to a behavioral change, not just gaining new knowledge. This theme was coded 19 times across the interviews. Participants describe how their attitudes towards the medical field changed over time in the program:

It got me interested [in] physician adjacent careers. Like research, because I never really thought about research, I never thought about becoming a scientist or anything like that, but through Lang and its affiliations with other programs, I was [exposed to] guest speakers [who are] doctors but they're also doing research… That was something that constantly came up where these people were doctors but they had so many other things going on and so many other things they were working on, and it really opened my eyes to different routes you can take even once you are a physician, and I think that also opened me up to getting my Master's in Public Health because I'm interested in the research side of medicine. (P5)

I always wanted to be a nurse. [Lang] opened my eyes to maybe [not wanting to be] a nurse… [Lang] opened my eyes to [other careers], maybe I could do something else not as clinical and still make an impact, and still be in the community. (P11)

#### Developing sense of self

Another intangible program driver found in the interviews was developing a sense of self. This was coded three times and defined as “Ways the program fostered participant intrinsic motivation for change and growth.” Participants describe how the program contributed to this development:

[Participating in the program] motivated me to find a balance between work and play… We joked around and had fun, but at the end of the day we had to remain very professional, [Lang] helped me shape my professional and personal self. And then tie those together without being ingenuine in the professional setting, but also being myself. (P21)

#### Did not pursue a health career

For some participants, a health career was not pursued for any number of reasons. Two participants in particular out of the 27 knew from the beginning that they were not interested in pursuing a career in medicine, but instead went through with the program for benefits such as scholarship money:

I took what was interesting to me, what was gratifying rather an encyclopedia for how to become a medical student, or, you know, I wasn't on that line. I was kind of just like riding the wave for my scholarship money. (P2)

While the majority of scholars had a genuine interest in medicine and health related careers, the resources and upward mobility associated with scholarships to pursue higher education was enticing to some.

#### Lang values

One of the most consistent intangible drivers of participant success was the internalization and enactment of specific values LYMP sought to distill onto students. Lang values were coded 9 times across the interviews and offered a rich perspective on how these values are passed from one generation to the next:

Their values. The values that [Lang] taught me I still use to this day. Respect. Compassion. Patience. Professionalism. Those are things that they instilled in us. Those are things that you learn and are hard to let go because it’s very important for the future. For you and who you become as a person. I remember a few weeks ago, I was working, and we had a volunteer and ironically, she’s a part of the [program]. I started speaking to her and she was on the younger side. And I was giving her advice: “Listen you might not see it now because you’re young and you want to have fun but…you’re going to be super grateful for it in the future. So, stay still, do your studies, commit to the summertime here. And in a few years, you’ll see the outcome of it. (P11)

### Improvement opportunities

While the Lang Youth Medical Program aims to foster an inclusive and supportive learning environment for all program participants, there are multiple areas that can be improved to enhance the program experience for future participants.

#### Program limitations

A number of participants gave specific critiques regarding program support, time commitments, program diversity and inclusivity, college preparation, and career choice. Five participants believe the program provided inconsistent or insufficient support resulting in a lack of sense of community and belonging. Some participants mentioned they never felt engaged in the alumni network and hoped Lang could create more feasible opportunities for them to connect with their fellow alumni.

Participants provided mixed comments on program academic content and parent engagement. While some participants believed the program should be more health-focused to prepare students for a pre-med track, others thought the program could incorporate more public health-relevant topics to broaden students’ perspectives and knowledge. In terms of parent engagement, one person believes the program has asked too much from parents, but another participant would like to see more parental involvement.

The Lang program was also criticized because some alumni think the program failed to provide a comprehensive image of pursuing health as a career. Some alumni found such an environment gave them an optimistic image of pursuing medicine as a career. Once they enter college, they “realize how expensive and how hard it is to become a doctor and kind of lose that vision and that optimism” (P4).

Other critiques include two alumni who expressed their disappointment towards the program because they were unable to secure a career through the Lang program to become Lang staff. While the program does not guarantee employment for participants post-graduation, these two participants felt disappointed as they were eager to make a contribution to the Lang community.

#### Recommendations

Study participants provided a wide range of thoughtful program recommendations in their interviews. Some of the recommendations were made specifically on the program’s approach to addressing career choices, program activities, program selection criteria, and program outreach efforts.

Several participants suggested that the program should be open to non-medicine-related career paths. They believe the program should consider “that not every student wants to be a doctor” (P21) and students “do not necessarily have to be a nurse or a doctor” (P11) to serve their community.

Recommendations were also given concerning program activities. A few participants recommended having the retreat at a different time of the year to give students more time to make friends before any group activities. Additionally, some alumni wished there were more outdoor activities including trips and outdoor lessons as “being in that open environment really helps [when studying]” (P18).

In terms of program selection criteria, a few participants indicated that they were never inclined to pursue medicine but were still accepted to the program. The program gave them a unique perspective but not necessarily an enjoyable experience throughout. One alumni mentioned that “at first, everything was really scary for me. And then it got scarier. And I think it stayed, it reminded me that I cannot work in the medical field because that is so scary for me. And it’s so much responsibility that I was not able to handle but it was so great to see it like a firsthand account” (P22).

Additionally, some participants recommended the program improve its outreach effort by conducting it “more frequently” (P6) and providing more relevant messages to young students (P1).

### External facilitators and barriers to program participation

One key area of interest to this evaluation was participants’ ability to fully participate in the program. While plenty of support was provided by the program to ensure this, interviews showed that sometimes superseding conditions and external forces affected participants’ ability to succeed or struggle. This topic area of external facilitators and barriers to program participation is divided into two major topic areas: external drivers and financial hardship.

#### External drivers

External drivers were defined as “any external factors outside of the Lang program that had an influence on a participant’s personal and/or professional decision making (e.g., family, work, money).” This theme was divided into two sub themes which researchers used as codes when analyzing interviews. The first code, external influences, was coded 7 times across the interviews and the second code was parental support, which was coded 9 times across the 27 interviews.

Oftentimes external influences provided a means of facilitating fuller participation in the program. For instance, having an older sibling who participated in the program was a major facilitator:

I’d say it made it a lot easier for me to adjust to the way the program would work. [Because] I know that I probably had an easier time than some of my peers who were just going through the program kind of confused and not knowing what would happen throughout the year. So, with my sister, I’d be able to like, hey do you know what happened during this year of your time in the program, and she’d let me know, like, we covered this stuff, and then I’d have a little bit of input on how the year would go. So, I think that made it a lot more simple in my eyes. (P23)

Others relied on parents who had experience in the medical field or who supported and pushed them to continue in the program:

I’d say [I] developed [an interest in health careers] very significantly… I always had a view -- I guess an informed view of medicine and stuff because even though I was little, my parents worked -- like they operated a hospital in Pakistan. And I’d often visit them, so it wasn’t like a really foreign field to me…but like I was able to be exposed to [the medical field] way earlier on, and for a while and with consistent help from that was really nice. (P6)

Yeah, definitely, it was—my mom probably helped out the most. My dad too, my dad would be there, um, he would come to like my expos and things like that, um, he would pick me up, he would stay late if he had to and things like that. And then my mom was like there in the morning, she’d be like, “Okay, get up, get your white coat on, get your gold pin, your ID and everything, make sure you have everything, let me make you breakfast,” things like that. And then if I ever was, like, doubting, like I said before, if I ever had any doubts about, like, going into medicine or things like that, she would just calmly, like, “Calm down, sit, let’s think about it, let’s talk about it,” things like that. (P18)

Still others’ success in the program was driven by external teachers who had facilitated participation in the program:

I was just…very lucky to always have had great teachers from kindergarten up until high school, and of course I have great professors as well, but I was really lucky to go to some schools that had teachers who really, really cared about the students. And so, my science teacher, she was the one who kind of like vetted who would go into the program or not--well, who would apply for the program. And she saw something within me, thinking that I would be a good candidate for the program. And I submitted an application and got the first few rounds of interviews, and I eventually got into the program. (P12)

#### Financial hardship

Financial hardship was defined as any “financial barriers that prevented a participant from participating in program activities or pursuing academic or professional goals.” This code was used four times across the interviews.

While many students found facilitators to success in these external influences, others spoke about the external barriers they or others faced to being entirely successful in the program.

Not all scholars lived within walking distance of the program. Most take the train and obviously your school pass only works Monday through Friday and so while I did not think it was a hardship at the time, I would imagine either based on my family income or whatever budgets there were, there always had to be some budget going towards transportation, clothes, and lunch. So, in retrospect, those were the things I thought about. (P10)

Other students spoke about the financially daunting prospect of attending medical school -- something that was encouraged as an ideal outcome by the program, but not feasible in the eyes of participants:

And I just knew, I guess I kind of knew from the outset that I probably wasn’t going to medical school, because it’s just, it’s way out there you know this commitment was enough for me. And also, financially, I wouldn’t have been able to afford it. (P2)

## Discussion

The Lang Youth Medical Programs seeks to increase the number of minority students who successfully complete health sciences related academic tracks and go on to pursue a profession in healthcare. Recognizing how challenging these developmental years may be and understanding that there are external drivers that influence student decision-making, LYMP has also embedded in its logic model ways to support students’ psychosocial health and overall well-being. To this end, the program immerses scholars in activities that focus on academic content, interpersonal and professional skills, goal setting, and exposes students to a variety of healthcare professions. This study sought to examine how the Lang Youth Medical Program impacted the academic and career paths of graduating scholars.

Participant responses demonstrate that the structure of the LYMP program is aligned with multiple youth development frameworks, such as the UNICEF Conceptual Framework for Measuring Outcomes of Adolescent Participation (26), and The Positive Youth Development, and the Development Assets frameworks (27, 28). These frameworks which provide guidance on what foundational knowledge and skills must be fostered for adolescent and young adults to achieve their personal and professional goals. Responses showcased ways in which the program demonstrated success of its desired outcomes — such as attainment of academic skills or pursuit of a health career — but also revealed facilitators of student success, including social and psychological reinforcement. Alumni described how being admitted to the program in the first place helped them see themselves as smart and capable, and how opportunities such as hospital visits and health science internships reinforced a sense of their belonging in the field. In some cases, the self-confidence fostered by the program helped participants transform interactions with program staff, internship coordinators, and coworkers into longer-term mentor relationships that provided them with support and social capital as they navigated life after the program. These results reinforce similar findings of previous studies that empowering youth to build mentorship relationships is an important pathway to post-high school academic and career success, particularly for youth from underrepresented minorities (33, 34). Additionally, alumni often expressed a desire to give back to the program and to their broader community, which is not a core component of the program, but an outcome developed through the reinforcement of programming focused on efficacy, capacity, and self-confidence building. Finally, the program equipped participants with life skills beyond the academic/hospital environment; for example, coping skills proved instrumental in helping participants navigate changes in their lives due to unforeseen events like the pandemic.

To better serve prospective and enrolled students, evaluation of the program should occur more frequently to ensure student/alumni feedback is integrated into the evolution of program activities. Such efforts can lead to more timely improvements to address program weaknesses and reduce unnecessary barriers for students to participate in the Lang program. For example, in response to some study participants expressing that the inability to pay for transportation related costs had prevented them from attending certain activities, program leadership has secured transportation subsidies.

Future research seeking to understand the impact the Lang Program has on scholars and alumni would be enhanced by qualitatively and quantitatively examining youth development constructs inherent in the program design, identifying a program enrollment time that would generate the greatest success, general educational and career outcomes of participants, and the perspectives of the professionals advancing the mission of the program by providing their time and expertise.

### Strengths and limitations

Our study has several key strengths. First, this study makes an important contribution to the literature by documenting the perspectives of how a health science professions pipeline program for minority students impacts the personal and career trajectories of program graduates who are entering or in the workforce. The Lang Program has implemented a unique health professions pipeline model, with this study being the first of its kind illustrating the key components of the program that past scholars found to be most beneficial to achieving their goals. Further, our qualitative inquiry facilitated a more detailed and nuanced exploration of our research question than would be captured by quantitative methods. As a result, themes and topic areas were identified organically during data analysis. Additionally, this study presents invaluable information about the kinds of external factors faced by minority students in reaching their goals, providing a call to action for other institutions to address systemic barriers.

There are a couple limitations to be noted. First, the interview tool was created from scratch by the research team and guided by the logic model. Development frameworks were used to consider what potential themes could arise, but the guide was not developed to test any specific development framework or construct. Second, given that many alumni graduated several years prior to the interview, responses could have been influenced by recall bias. However, this also allowed participants to provide feedback over a longer time period. Thirdly, selection bias may also be present, as the research team did not have accurate contact information for all eligible alumni. Participants whose information was up-to-date and participated in the study may be more engaged with the program than non-participants who could not be reached. Although the research team did not seek to generalize findings from this study to the entire student population, self-selection from this convenience sample may result in biased perspectives. Fourthly, 27 of the anticipated 30 interviews were completed, which may have left certain perspectives undiscovered. Although the research team found that data and thematic saturation was met for the collected sample, our sample is different from the program participant population in terms of race/ethnicity and gender. Not being able to interview more alumni could result in missing themes, but such limitation is not unexpected given this is a convenience sample. Lastly, alumni understand the mission of the program, and many have an interest in seeing the program succeed, therefore, it is possible that responses were primed by social desirability bias.

### Implications

Design principles from the program (see Appendix 2) and findings from this study can be used by health care systems looking to increase the number of underrepresented minority youth seeking careers in the health sciences. Specifically, ways by which to invest educational and professional development resources into their local community. Adopting the same program structure in other communities to address the gaps in educational resources availability and accessibility can be transformative, with lasting impact far into the future and across all aspects of a participant’s life. However, replication of Lang’s program success requires organizations to be explicit in who their target audience is, and the program should be made to meet their needs.

## Data availability statement

The original contributions presented in the study are included in the article/Supplementary material, further inquiries can be directed to the corresponding author.

## Ethics statement

The studies involving humans were approved by Columbia University Human Research Protection Office and IRBs. The studies were conducted in accordance with the local legislation and institutional requirements. The participants provided their written informed consent to participate in this study.

## Author contributions

OK, ZW, and RS contributed to the conception and design of the study. The same three conducted the analysis and interpretation of the data, and draft of the initial manuscript. OK, ZW, RS, MM, and JR contributed to revisions of the paper. AN provided the finwal revisions and approved the submission of the paper. OK and ZW contributed equally to this work. All authors contributed to the article and approved the submitted version.
