# Supplementary material for: Assessing the impact of a 6-year health sciences enrichment program for underrepresented minority youth on healthcare workforce diversity, career path, and public health
Source: Front Public Health. 2023 Oct 12;11:1244593. doi: 10.3389/fpubh.2023.1244593 (PMC10602669; doi:10.3389/fpubh.2023.1244593)
Supplement: Supplementary file 1 [file Data_Sheet_1.PDF]

NewYork-Presbyterian Inspiring Possibilities **Lang Youth Medical Program**

Curriculum Overview

The Lang Youth Medical Program provides a robust overview of the human body from medical and public health perspectives. Each semester is dedicated to a specific body system, following the same lesson order:

1. Body System Introduction
2. Pathologies
3. Diagnosis & Treatment
4. Careers
5. Community Health
6. Global Health

Body System Curriculum Schedule

7th Grade

- Fall – Introduction to the Healthy Human Body
- Spring – Cardiovascular System

8th Grade

- Summer – Environmental Medicine & Epidemiology
- Fall – Endocrine System
- Spring – Integumentary System

9th Grade

- Summer – Digestive System
- Fall – Immune System
- Spring – Musculoskeletal System

10th Grade

- Fall – Renal System
- Spring – Genetics

11th Grade

- Fall – Reproductive System
- Spring – Respiratory System

12th Grade

- Summer/Fall – College Preparation
- Spring – Nervous System

Program Activities & Objectives

Hospital Tours

- Understand the various departments, functions, and workflows within a hospital setting.
- Gain insight into the role of different healthcare professionals and their collaboration in patient care.

Career Panels

- Explore diverse healthcare career pathways and specialties available within the medical field.
- Engage in discussions with experienced professionals to learn about their journeys, challenges, and successes.
- Identify key skills, educational requirements, and qualities essential for different healthcare roles.

Paid Internships in Clinical and Non-clinical Health Fields

- Understand the day-to-day responsibilities of different roles, such as nursing, administration, research, or medical technology.

Personal and Professional Skills development workshops.

- Cultivate leadership qualities, teamwork, and ethical decision-making skills in the context of healthcare.

Classes on Human Body Systems:

- Grasp human anatomy, functions, and interactions of body systems.
- Recognize disease implications and apply knowledge in healthcare.

Disease Pathology and Prevention:

- Study disease causes, progression, and preventive strategies.
- Explore public health interventions and lifestyle impacts.

Case Study Round Tables:

- Develop critical thinking via patient case analysis.

Global and Public Health Curriculums:

- Understand wider health contexts, disparities, and factors.
- Learn about global health challenges and community engagement.

College Application Assistance

- Equip students with the skills to craft compelling college applications that showcase their achievements, aspirations, and unique qualities, enhancing their chances of admission to higher education institutions.

SAT Tutoring

- Provide targeted instruction and strategies to help students improve their SAT scores.

College Campus Tours

- Foster informed decision-making by exposing students to various college environments and facilities, helping them assess compatibility and make well-informed choices about their higher education journey.

Alumni Career Development

- Facilitate networking opportunities, mentorship, and career guidance through alumni connections, empowering students to make informed career decisions and successfully transition from college to their chosen professional paths.

Exposure to and Guidance from Healthcare Professionals, and Students from Undergraduate, Graduate, and Professional Schools

- Enhance participants' understanding of healthcare professions by facilitating direct interactions and mentorship with a diverse range of healthcare professionals, as well as students at different educational levels (undergraduate, graduate, and professional), providing valuable insights into various healthcare career paths, educational journeys, and practical experiences.

Connecting Students and Families to Community Healthcare and Wellness Services

- Facilitate access to essential community health care and wellness services by connecting students and their families, thereby increasing awareness, utilization, and understanding of available resources, ultimately promoting overall health and well-being within the community.
